# Role of Cyclin B1/Cdc2 Up-Regulation in the Development of Mitotic Prometaphase Arrest in Human Breast Cancer Cells Treated with Nocodazole

**DOI:** 10.1371/journal.pone.0024312

**Published:** 2011-08-30

**Authors:** Hye Joung Choi, Masayuki Fukui, Bao Ting Zhu

**Affiliations:** Department of Pharmacology, Toxicology and Therapeutics, School of Medicine, University of Kansas Medical Center, Kansas City, Kansas, United States of America; Virginia Commonwealth University, United States of America

## Abstract

**Background:**

During a normal cell cycle, the transition from G_2_ phase to mitotic phase is triggered by the activation of the cyclin B1-dependent Cdc2 kinase. Here we report our finding that treatment of MCF-7 human breast cancer cells with nocodazole, a prototypic microtubule inhibitor, results in strong up-regulation of cyclin B1 and Cdc2 levels, and their increases are required for the development of mitotic prometaphase arrest and characteristic phenotypes.

**Methodology/Principal Findings:**

It was observed that there was a time-dependent early increase in cyclin B1 and Cdc2 protein levels (peaking between 12 and 24 h post treatment), and their levels started to decline after the initial increase. This early up-regulation of cyclin B1 and Cdc2 closely matched in timing the nocodazole-induced mitotic prometaphase arrest. Selective knockdown of cyclin B1or Cdc2 each abrogated nocodazole-induced accumulation of prometaphase cells. The nocodazole-induced prometaphase arrest was also abrogated by pre-treatment of cells with roscovitine, an inhibitor of cyclin-dependent kinases, or with cycloheximide, a protein synthesis inhibitor that was found to suppress cyclin B1 and Cdc2 up-regulation. In addition, we found that MAD2 knockdown abrogated nocodazole-induced accumulation of cyclin B1 and Cdc2 proteins, which was accompanied by an attenuation of nocodazole-induced prometaphase arrest.

**Conclusions/Significance:**

These observations demonstrate that the strong early up-regulation of cyclin B1 and Cdc2 contributes critically to the rapid and selective accumulation of prometaphase-arrested cells, a phenomenon associated with exposure to microtubule inhibitors.

## Introduction

Nocodazole, a prototypic microtubule inhibitor [1], [2], has anticancer activity and is also widely used in cell biology research as a tool for synchronization of the cell division cycle [3]–[6]. Mechanistically, this chemical can bind to tubulins and microtubules, thereby suppressing microtubule dynamics [7]. Disruption of microtubule formation and function in cells treated with nocodazole [8], [9] or other microtubule inhibitors (*e.g.*, vinblastine, colchicine, and paclitaxel) [9], [10]–[12] results in suppression of cell cycle progression, with cells usually arrested in the G_2_/M phase (based on flow cytometric analysis of cellular DNA content).

During a normal cell cycle, the progression of cells in the G_2_ phase to M phase is triggered by the activation of the cyclin B1-dependent Cdc2 kinase [13]–[15], which is regulated by a series of phosphorylation-dephosphorylation events and protein-protein interactions [16]–[19]. In general, a cell with a suppressed cyclin B1/Cdc2 activity would tend to be arrested in the G_2_ phase, whereas a cell with an elevated cyclin B1/Cdc2 activity would be favored to enter mitosis [20]. This general principle is supported by a large body of experimental observations. For instance, earlier studies showed that treatment of cells with roscovitine, an inhibitor of the cyclin-dependent kinases (CDKs), or selective knockdown of cyclin B and Cdc2 expression with siRNAs, each produced cell cycle arrest predominantly in the G_2_ phase, with a simultaneous reduction of cells in the M phase [21]. However, during the induction of the G_2_/M phase cell cycle arrest (based on analysis of cellular DNA content) following treatment of cells with microtubule inhibitors such as nocodazole and paclitaxel, it has been observed in some earlier studies that there was a marked increase in cyclin B1 and Cdc2 protein levels [22]–[24]. When the morphology of the cells treated with microtubule inhibitors was analyzed, it was found that most of the G_2_/M cell population were actually arrested in mitotic prometaphase, but not in the G_2_ phase [25]. The functional role of this puzzling strong increase in cyclin B1 and Cdc2 protein levels in the development of mitotic prometaphase arrest in cells treated with microtubule inhibitors is not understood at present, which was the focus of our present investigation. Using nocodazole, a prototypic microtubule inhibitor, as a tool drug, here we performed a series of experiments demonstrating that the strong early up-regulation of cyclin B1/Cdc2 contributes critically to the rapid accumulation of cells selectively arrested in the mitotic prometaphase. In addition, we found that the mitotic arrest deficient 2 (MAD2) protein, an important spindle checkpoint protein, is involved in mediating nocodazole-induced up-regulation of cyclin B1 and Cdc2.

## Results

### Characterization of nocodazole-induced mitotic arrest

**Figure 1:**
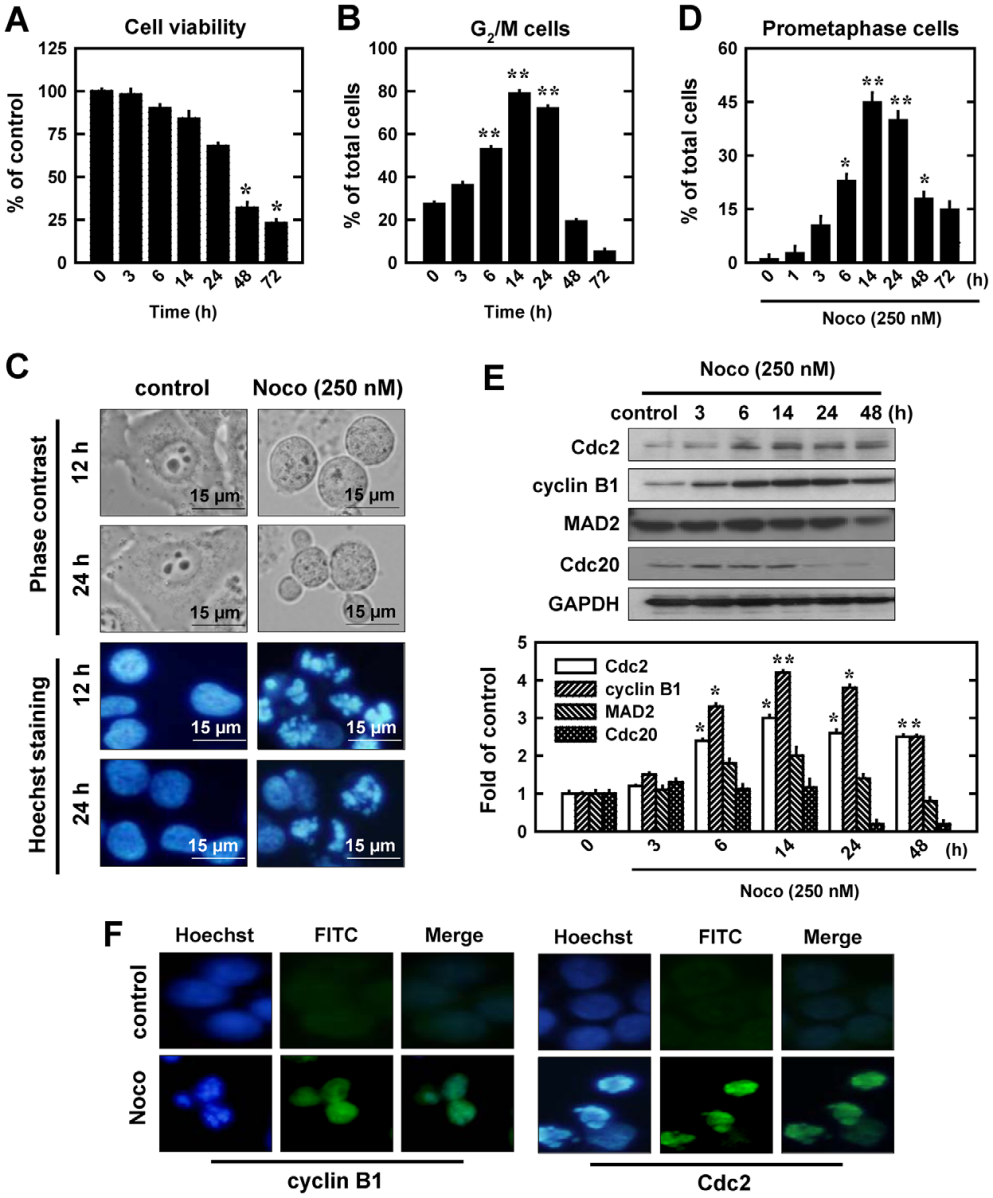
Induction of cell cycle arrest and cyclin B1/Cdc2 activation in MCF-7 cells by nocodazole (Noco). **A**. Changes in cell viability (MTT assay) after treatment of cells with 250 nM nocodazole for different lengths of time. Each data point is the mean ± S.D. from four replicate measurements from one representative experiment. **B**. Time-dependent induction of G_2_/M cell cycle arrest following treatment with nocodazole. Cells were seeded at 5×10^4^ cells/mL and then treated with 250 nM nocodazole for 3, 6, 14, 24, 48 and 72 h. Cells were harvested and analyzed using flow cytometry. **C**. Cells were treatment with 250 nM nocodazole for 12 or 24 h, stained with Hoechst-33342, and examined in a phase contrast (PC) microscope (upper panel) or a fluorescence microscopy (lower panel) (at ×200 magnification). As shown, many cells are arrested in mitosis (prometaphase) after 250 nM treatment. **D**. Cells were treated with 250 nM nocodazole for 3, 6, 14, 24, 48 and 72 h. The morphology of cells arrested in prometaphase (based on 200 or more nuclei in each sample) was scored by fluorescence microscopy. Each bar is the mean ± S.D. value from three separate experiments. * *P*<0.05, ** *P*<0.01 versus vehicle-treated control. **E** (**upper part**). Time-dependent changes in cyclin B1 and Cdc2 protein levels following nocodazole treatment. Cells were treated with nocodazole (250 nM) for the length of time as indicated, and whole cell lysates were prepared. An equal amount of protein lysates was electrophoretically separated on the 10% SDS-polyacrylamide gel, and transferred to nitrocellulose membrane. Western blots were detected using specific antibodies against cyclin B1, Cdc2 (CDK1), MAD2, and Cdc20 on an enhanced chemiluminescence (ECL) apparatus. Membrane was stripped for determining the levels of GAPDH as a loading control. **E** (**lower part**). The relative protein levels for cyclin B1, Cdc2, MAD2, and Cdc20 were calculated according to their densitometry readings, which were normalized according to the corresponding readings for the GAPDH protein bands. Each value is mean ± S.D. from three replicate determinations. * *P*<0.05, ** *P*<0.01 versus vehicle-treated control. **F**. Cells were treatment with 250 nM nocodazole for 12 h and analyzed using immunofluorescence staining for cyclin B1 and Cdc2. Representative photographs were taken under a fluorescence microscope (original magnification, ×200).

Exposure of MCF-7 human breast cancer cells to nocodazole reduced cell viability (MTT assay) in a concentration- and time-dependent manner (**Figure 1A**). Flow cytometry analysis showed that nocodazole (250 nM) induced characteristic G_2_/M-pattern cell cycle arrest, starting as early as at 3 h after drug treatment and reached plateau at 14 h (79% compared to 27.3% in control cells) (**Figure 1B**). Analyses of the nuclear morphology showed that nocodazole-treated cells exhibited chromosomal condensation and segregation (**Figure 1C**), which are characteristic morphological changes seen in cells blocked in mitotic prometaphase [26]. Based on counting the number of prometaphase cells (*i.e.*, the mitotic index), the time-dependent change in nocodazole-induced mitotic prometaphase arrest (**Figure 1D**) matched closely the change in the combined G_2_/M cell population (**Figure 1B**). Together, these data suggest that nocodazole predominantly induces mitotic prometaphase arrest in MCF-7 human breast cancer cells in a time- and concentration-dependent manner.

As shown in **Figure S1A** and **S1B**, the induction of prometaphase arrest was also observed in another human breast cancer cell line (namely, the ER-negative MDA-MB-435s cells) following *in vitro* treatment with nocodazole for 12 or 24 h. Similarly, the induction of prometaphase arrest by nocodazole was also observed in MCF-10A cells (a non-tumorigenic human mammary epithelial cell line) (**Figure S2B** and **S2C**). It appears that MCF-10A cells are more sensitive to the induction of cell death by nocodazole (data not shown), likely due to the faster proliferation rate of MCF-10A cells compared to MCF-7 cells (**Figure S2A**).

In all three human cell lines tested in this study, we found that the mitotic arrest induced by nocodazole was associated with a marked up-regulation of cyclin B1 and Cdc2 protein levels (**Figure 1E** **, Figure S1C, Figure S2D**). This finding confirms earlier observations with other antitubulin agents [20], [27]. Using MCF-7 cells as a representative model, we further conducted detailed time-course analysis of the levels of these two cell cycle proteins. Their levels started to increase at 3 h after nocodazole treatment and reached a peak between 14 and 24 h, but after the initial 24 h, their levels were markedly decreased in a time-dependent manner (**Figure 1E**). It is of note that the time-dependent increase in cyclin B1 and Cdc2 levels following nocodazole treatment closely mirrored the time-dependent induction of prometaphase arrest (compare **Figure 1D and 1E**). Moreover, the magnitude of the increase in cyclin B1 and Cdc2 protein levels and the severity of prometaphase arrest depended on the concentrations of nocodazole used; in general, a stronger up-regulation of these two proteins and a greater severity of prometaphase arrest were seen when higher concentrations of nocodazole were present (data not shown).

### Role of cyclin B1 and Cdc2 in the development of mitotic prometaphase arrest

Accumulation of cyclin B1 and Cdc2 in the nucleus of a cell is known to trigger the development of chromosomal condensation and segregation, which are characteristic morphological changes seen in cells blocked in prometaphase [25]. To probe whether the early up-regulation of cyclin B1 and Cdc2 protein levels contributed to the observed nuclear morphological changes in nocodazole-treated cells, we first examined the subcellular localization of these two proteins in control and nocodazole-treated cells using the immunofluorescence staining approach. As shown in **Figure 1F**, while the levels of these two proteins were very low in both cytosol and nuclei of untreated control cells, their levels were drastically and selectively increased in the nuclear compartment of nocodazole-treated cells. This observation suggests that during the induction of mitotic prometaphase arrest by nocodazole, there is a marked nuclear accumulation of these two cell cycle-regulatory proteins.

**Figure 2:**
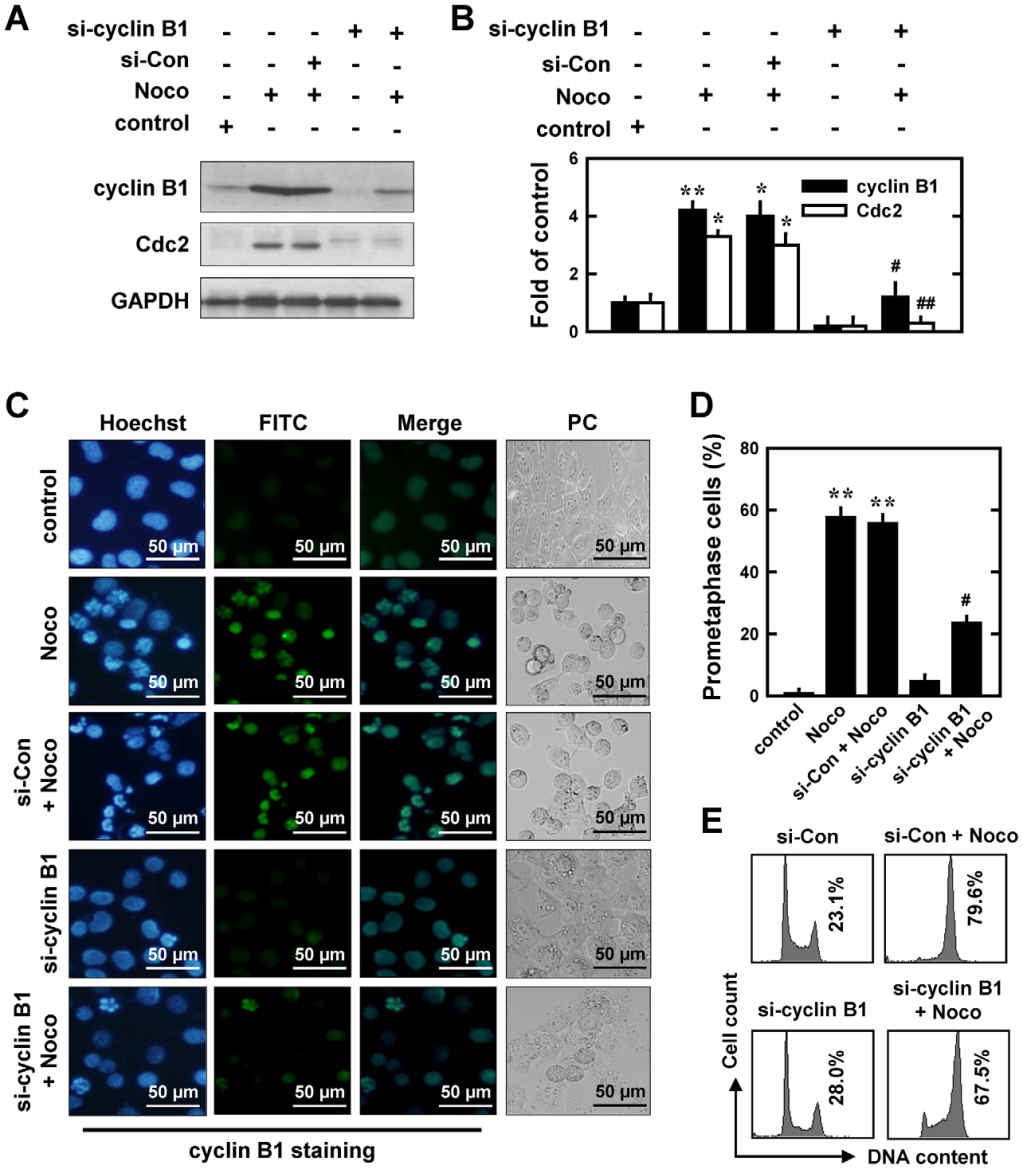
Effect of cyclin B1 knockdown on the development of nocodazole (Noco)-induced prometaphase arrest in MCF-7 cells. **A**. Cells were transfected with cyclin B1 siRNA (si-cyclin B1) and the negative control siRNAs (si-Con), 24 h later, cells were exposed to 250 nM nocodazole for additional 12 h. Then the whole cell lysates were analyzed for the levels of cyclin B1 and Cdc2by Western immunoblotting. **B**. Relative protein levels of cyclin B1 and Cdc2 are calculated according to densitometry readings, which are then normalized according to the corresponding readings for the GAPDH protein bands. Each value is mean ± S.D. from three replicate determinations. * *P*<0.05, ** *P*<0.01 versus vehicle-treated control; ^#^ *P*<0.05, ^##^ *P*<0.01 versus nocodazole treatment. **C**. Cells were transfected with si-cyclin B1 or siRNA negative control and then further treated with nocodazole (250 nM) for 12 h. Cells with cyclin B1 knockdown were analyzed using immunofluorescent staining for cyclin B1. Representative photographs were taken using a fluorescence microscope (original magnification, ×200) or a phase contrast (PC) microscope (×200). **D**. Quantitative data on prometaphase-arrested cells. Each bar is a mean ± S.D. value from three separate experiments. * *P*<0.05, ** *P*<0.01 versus the vehicle-treated control; ^#^ *P*<0.05 versus nocodazole treatment. **E**. The DNA content of cells was analyzed using flow cytometry as described in the Material and Methods section.

To provide definitive experimental evidence for the involvement of cyclin B1 and Cdc2 up-regulation in nocodazole-induced prometaphase arrest, we employed the siRNA approach to selectively knock down the expression of cyclin B1, Cdc2, or both. As shown in **Figure 2A, 2B**, twenty-four h after transfection with cyclin B1-specific siRNA (si-cyclin B1), cells were treated with nocodazole and then harvested for Western blot analysis of cyclin B1 and Cdc2 levels. Knockdown of cyclin B1 abrogated nocodazole-induced increase in both cyclin B1 and Cdc2 proteins compared with control siRNA-transfected cells. Moreover, immunofluorescence staining using anti-cyclin B1 antibodies showed that transfection with si-cyclin B1 diminished nocodazole-induced nuclear accumulation of cyclin B1 protein (**Figure 2C**). These changes were accompanied by a reduction in the degree of chromosomal condensation and segregation, as well as a decrease in the population of prometaphase cells (from 58.0% to 23.7%, *P*<0.05) (**Figure 2D**). Notably, in cells with cyclin B1 knockdown, treatment with nocodazole caused a smaller increase in the combined G_2_/M cell population (assessed by flow cytometric analysis) (**Figure 2E**). These data indicate that cyclin B1 knockdown predominantly decreases the population of prometaphase cells while it only slightly reduces the combined G_2_/M cell population.

**Figure 3:**
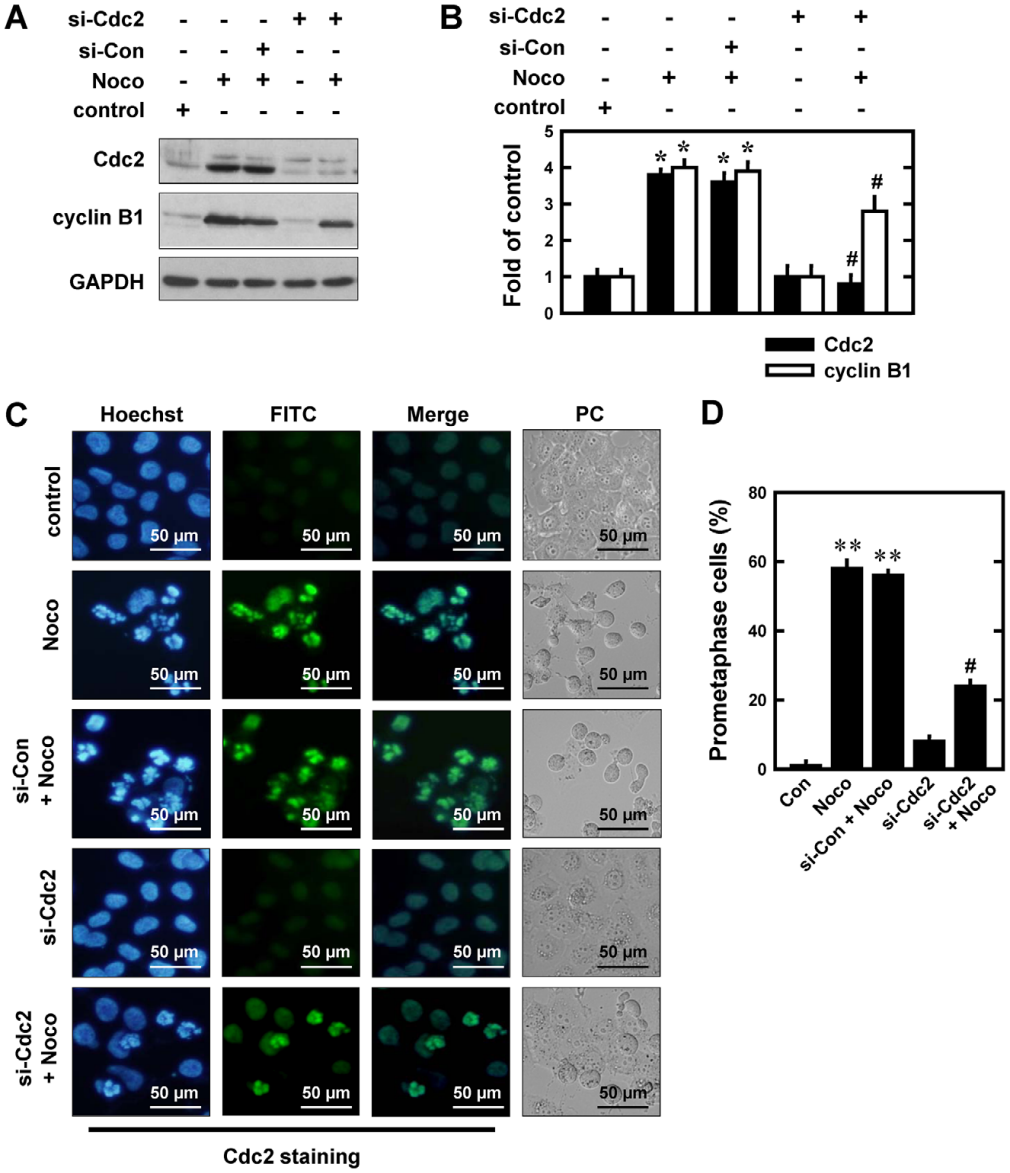
Effect of Cdc2 knockdown on the development of nocodazole (Noco)-induced prometaphase arrest in MCF-7 cells. **A**. Cells were transfected with siRNA Cdc2 (si-Cdc2) and the negative control siRNAs (si-Con), and 24 h later, cells were exposed to 250 nM nocodazole for additional 12 h. Then the whole cell lysates were analyzed for Cdc2 and cyclin B1 levels using Western immunoblotting. **B**. The relative protein levels of Cdc2 and cyclin B1 are calculated according to their densitometry readings, which are normalized according to the corresponding readings for the GAPDH protein bands. Each value is mean ± S.D. from three replicate measurements. * *P*<0.05 versus vehicle-treated control; ^#^ *P*<0.05 versus nocodazole treatment. **C**. Cells were transfected with si-cyclin B1 or siRNA negative control and then further treated with nocodazole (250 nM) for 12 h. Cells with Cdc2 knockdown were analyzed using immunofluorescent staining. Representative photographs were taken using a fluorescence microscopy (original magnification, ×200) or a phase contrast microscope (×200). **D**. Quantitative data on prometaphase-arrested cells. Each bar is a mean ± S.D. value from three separate experiments. ** *P*<0.01 versus the vehicle-treated control; ^##^ *P*<0.01 versus nocodazole treatment.

Similarly, knockdown of Cdc2 abrogated the nocodazole-induced accumulation of Cdc2, but the cyclin B1 level was only modestly reduced in comparison with the control siRNA-transfected cells (**Figure 3A, 3B**). Immunofluorescence staining using anti-Cdc2 antibodies showed that transfection with Cdc2 siRNAs significantly reduced nocodazole-induced nuclear accumulation of Cdc2 protein (**Figure 3C**). Similar to the observations with cyclin B1, knockdown of Cdc2 was associated with a reduction in prometaphase cells (from 58.0% to 24.0%) (**Figure 3D**). Collectively, these results suggest that the marked increase in cyclin B1 and Cdc2 protein levels contributes to the accumulation of prometaphase-blocked cells following nocodazole treatment. Following Cdc2 knockdown, the population of G_2_/M-arrested cells as assessed by flow cytometry analysis was only mildly reduced after nocodazole treatment (data not shown). These data indicate that knockdown of Cdc2, similar to what was observed with cyclin B1 knockdown, predominantly decreases the population of prometaphase cells while it affects the combined G_2_/M cell population to a much lesser degree.

### Role of MAD2 protein in mediating cyclin B1 and Cdc2 up-regulation and prometaphase arrest in nocodazole-treated cells

MAD2, an important spindle checkpoint protein, can block the progression through the metaphase-to-anaphase transition by binding to unattached kinetochores [28]. In addition, during the development of prometaphase arrest, this protein can also inhibit the activity of the anaphase promoting complex (APC) by sequestering Cdc20 until all chromosomes are attached by microtubules and properly aligned at the metaphase plate. To understand the role of MAD2 protein in mediating cyclin B1/Cdc2 up-regulation in nocodazole-treated cells, we first examined the time-dependent changes of MAD2 protein as well as a representative target protein, Cdc20, during nocodazole-induced cell cycle arrest. We found that the MAD2 protein level was only slightly increased between 6–14 h after nocodazole treatment, whereas Cdc20 protein level remained unchanged during the first 14 h and was markedly decreased at 24 and 48 h (**Figure 1E**).

**Figure 4:**
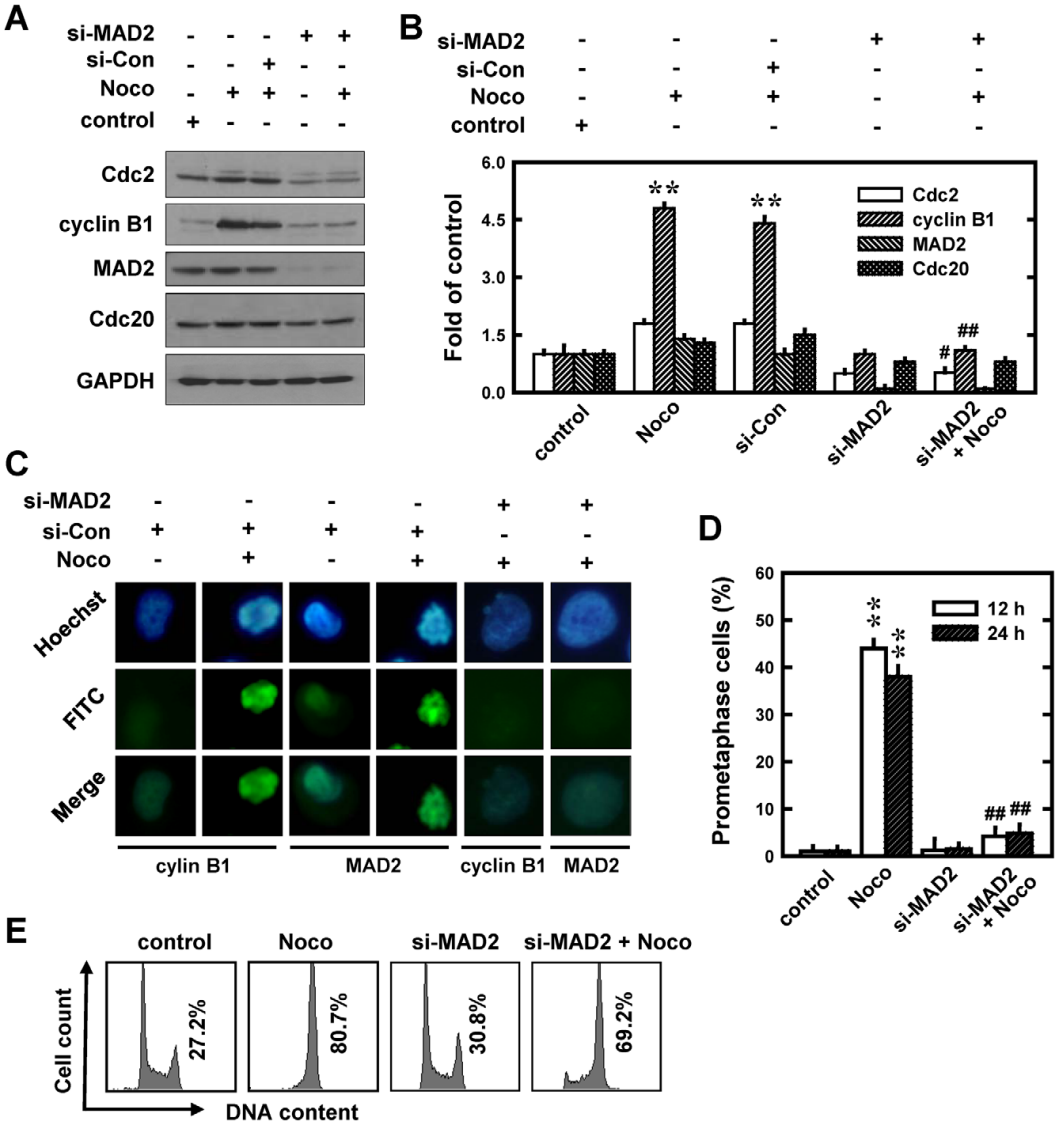
Effect of MAD2 knockdown on the development of nocodazole (Noco)-induced prometaphase arrest in MCF-7 cells. **A**. Cells were transfected with siRNA MAD2 (si-MAD2) and the negative control siRNAs (si-Con), and 24 h later, cells were exposed to 250 nM nocodazole for additional 12 h. Then the whole cell lysates were analyzed for Cdc2, cyclin B1, MAD2, and Cdc20 levels using Western immunoblotting. **B**. The relative protein levels of Cdc2, cyclin B1, MAD2, and Cdc20 are calculated according to their densitometry readings, which are normalized according to the corresponding readings for the GAPDH protein bands. Each value is mean ± S.D. from three replicate measurements. * *P*<0.05 versus vehicle-treated control; ^#^ *P*<0.05 versus nocodazole treatment. **C**. Cells were transfected with si-MAD2 or siRNA negative control and then further treated with nocodazole (250 nM) for 12 h. Cells with Cdc2 knockdown were analyzed using immunofluorescent staining. Representative photographs were taken using a fluorescence microscopy (original magnification, ×200). **D**. Quantitative data on prometaphase-arrested cells. Each bar is a mean ± S.D. value from three separate experiments. ** *P*<0.01 versus the vehicle-treated control; ^#^ *P*<0.05 versus nocodazole treatment. **E**. The DNA content of cells was analyzed using flow cytometry as described in the Material and Methods section.

Next, we selectively knocked down MAD2 expression and then studied its effect on nocodazole-induced changes in cyclin B1/Cdc2 levels as well as prometaphase arrest. As shown in **Figure 4A and 4B**, 24 h after transfection with the MAD2 siRNA (si-MAD2), cells were treated with nocodazole and then harvested for Western blotting of cyclin B1, Cdc2, MAD2, and Cdc20 protein levels. si-MAD2-transfected cells had a markedly reduced MAD2 protein level, suggesting that the knockdown was effective. Surprisingly, MAD2 knockdown completely abrogated the nocodazole-induced up-regulation of cyclin B1 and Cdc2 compared with control siRNA-transfected cells (**Figure 4A, 4B**). However, the level of Cdc20 was not appreciably altered in MAD2-knockdown cells treated with nocodazole (**Figure 4A, 4B**).

Immunofluorescence staining showed that cells with MAD2 knockdown had markedly reduced nuclear accumulation of cyclin B1 and MAD2 proteins following nocodazole treatment compared to cells transfected with control siRNA, which had highly elevated levels of nuclear cyclin B1 and MAD2 (**Figure 4C**). These changes were accompanied by a reduction in the degree of chromosomal condensation and segregation as well as a drastic decrease in the population of mitotic prometaphase cells from 44% to 4.2% (*P*<0.01) at 12 h and 38% to 4.8% (*P*<0.01) at 24 h (**Figure 4D**). Similar to what was observed with cyclin B1 or Cdc2 knockdown, knockdown of MAD2 only weakly affected the total cellular DNA content (*i.e.*, the combined G_2_/M cell population) in nocodazole-treated cells (**Figure 4E**). These results show that up-regulation of cyclin B1 and Cdc2 protein levels and the development of mitotic arrest both depend on the presence of MAD2 protein. Here it is also of note that MAD2 knockdown produced a stronger suppression of the prometaphase arrest than knockdown of cyclin B1 and Cdc2. This reason is because MAD2 knockdown will not only reduce the levels of cyclin B1 and Cdc2 (as shown in **Figure 4**), which reduces prometaphase arrest in the same way as does cyclin B1/Cdc2 knockdown, but it will also allow prometaphase cells to proceed through metaphase and then enter anaphase, which is an inherent function of MAD2.

### Pharmacological inhibition of Cdc2 abrogates nocodazole-induced prometaphase arrest

The series of experiments as described above suggested that up-regulation of cyclin B1/Cdc2 plays a critical role in the development of the characteristic prometaphase arrest seen in cells treated with microtubule inhibitors. This mechanistic explanation was put to further test by examining the effects on cell cycle changes when nocodazole was used in combination with rescorvitine, which is an inhibitor of the cyclin-dependent kinases (including Cdc2), or with cycloheximide (CHX), which is a protein sysnthesis inhibitor. The findings are briefly summarized below.

#### Effect of roscovitine

**Figure 5:**
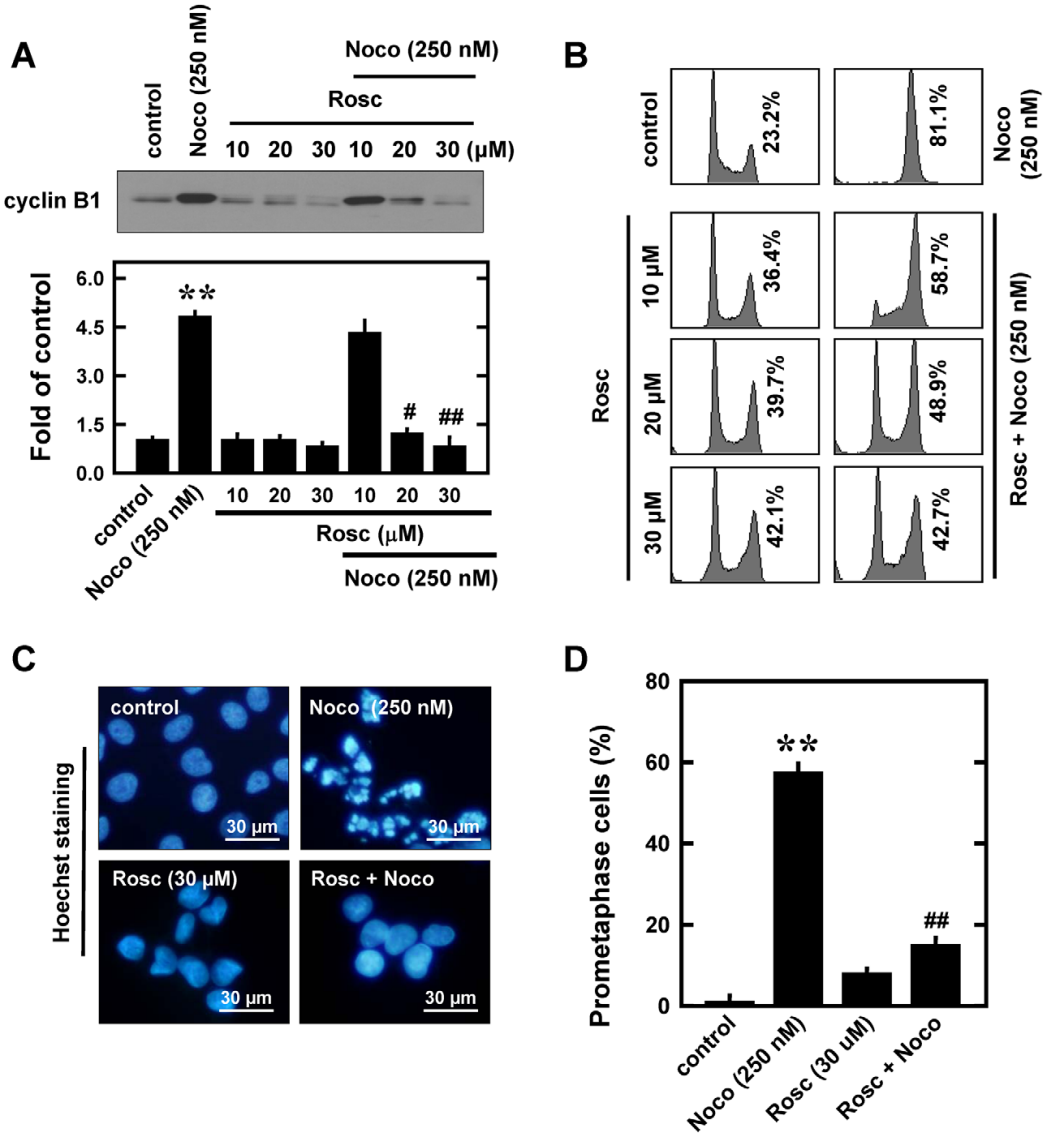
Effect of rescorvitine (Rosc) on nocodazole (Noco)-induced prometaphase arrest in MCF-7 cells. **A** (**upper panel**). Cells were pre-treated for 2 h with roscovitine (10, 20, and 30 µM) and then stimulated for additional 12 h with 250 nM nocodazole. Total cell lysates were analyzed by Western immunoblotting for cyclin B1. **A** (**lower panel**). The relative protein levels cyclin B1 are calculated according to their densitometry readings, which are normalized according to the corresponding readings for the GAPDH protein bands. Each value is mean ± S.D. from three replicate measurements. ** *P*<0.01 versus vehicle-treated control; ^#^ *P*<0.05, ^##^ *P*<0.01 versus nocodazole treatment. **B**. Cells were pre-treated for 2 h with roscovitine (10, 20, and 30 µM) and then stimulated for additional 12 h with 250 nM nocodazole. The DNA content of cells was analyzed using flow cytometry as described in the Material and Methods section. **C**. Nuclei were stained with Hoechst-33342, and examined using a fluorescence microscope for prometaphase cells (original magnification, ×200). **D**. Quantitative data on prometaphase-arrested cells. Each bar is a mean ± S.D. value from three separate experiments. ** *P*<0.01 versus the vehicle-treated control; ^##^ *P*<0.01 versus nocodazole treatment.

To study the modulating effect of roscovitine, MCF-7 cells were treated with 10, 20 and 30 µM roscovitine alone or in combination with 250 nM nocodazole for 12 h. Roscovitine (at 20 and 30 µM) markedly reduced nocodazole-induced increase in cyclin B1 levels (**Figure 5A**), and a similar reduction was seen with Cdc2 protein levels (data not shown).

Flow cytometric analysis of the cellular DNA content showed that treatment of cells with roscovitine alone increased the combined G_2_/M-arrested cells in a concentration-dependent manner, which is consistent with an earlier study [21]. During the development of roscovitine-induced G_2_/M arrest, the population of prometaphase cells is only slightly increased compared to cells treated with nocodazole (**Figure 5C**). Our observations are in agreement with the earlier study showing that roscovitine blocks MCF-7 cells in the G_2_ phase [21]. However, when roscovitine was used in combination with nocodazole, it strongly suppressed nocodazole-induced accumulation of the G_2_/M cell population (flow cytometric analysis, **Figure 5B**) as well as prometaphase-arrested cells (**Figure 5C, 5D**). The dose-dependent effect of roscovitine in suppressing nocodazole-induced accumulation of both G_2_/M and prometaphase cell populations is closely correlated with its ability to suppress cyclin B1 up-regulation in nocodazole-treated cells (**Figure 5**). Together, these data show that roscovitine, when used in combination with nocodazole, can inhibit cells from entering prometaphase by keeping them staying at the end of G_2_ phase. Mechanistically, roscovitine exerts this effect through inhibiting Cdc2 activity plus suppressing nocodazole-induced cyclin B1 up-regulation.

#### Effect of cycloheximide (CHX)

**Figure 6:**
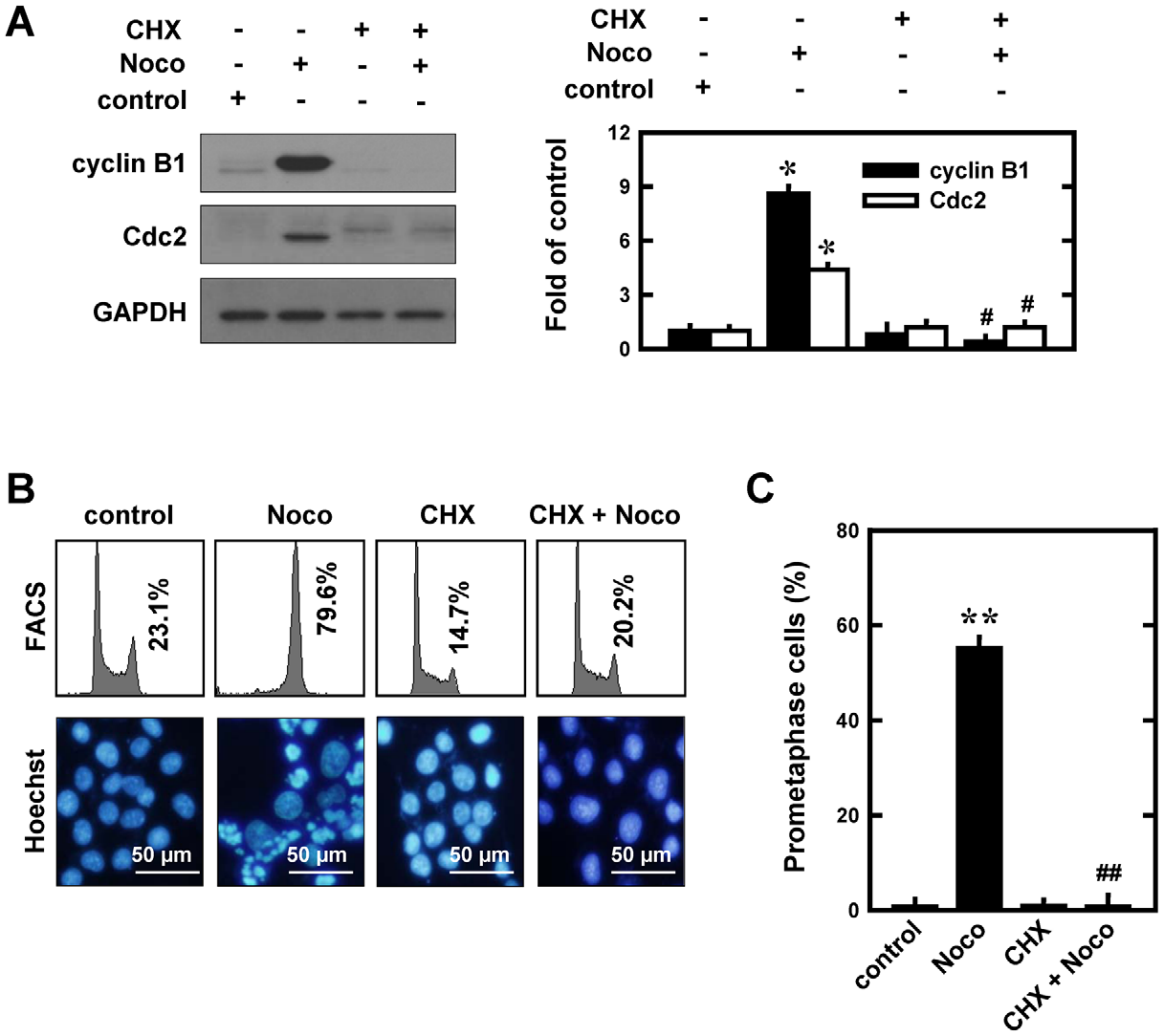
Effect of cycloheximide (CHX) on nocodazole-induced prometaphase arrest in MCF-7 cells. **A** (**left panel**). Cells were pre-treated for 2 h with cycloheximide (5 µg/mL) and then stimulated for additional 12 h with 250 nM nocodazole. Total cell lysates were analyzed by Western immunoblotting for cyclin B1 and Cdc2. **A** (**right panel**). The relative protein levels of cyclin B1 and Cdc2 are calculated according to their densitometry readings, which are normalized according to the corresponding readings for the GAPDH protein bands. Each value is mean ± S.D. from triplicate measurements. * *P*<0.05 versus vehicle-treated control; ^#^ *P*<0.05 versus nocodazole treatment. **B** (**upper panel**). Cells were pre-treated for 2 h with cycloheximiden (5 µg/mL) and then stimulated for additional 12 h with 250 nM nocodazole. The DNA content of cells was analyzed using flow cytometry as described in the Material and Methods section. **B** (**lower panel**). Nuclei were stained with Hoechst-33342, and examined using a fluorescence microscope for prometaphase cells (original magnification, ×200). **C**. Quantitative data on prometaphase-arrested cells. Each bar is a mean ± S.D. value from three separate experiments. ** *P*<0.01 versus the vehicle-treated control; ^##^ *P*<0.01 versus nocodazole treatment.

To investigate the modulating effect of CHX (a protein synthesis inhibitor) on nocodazole-induced cyclin B1/Cdc2 accumulation and mitotic arrest, MCF-7 cells were treated nocodazole alone or in combination with CHX (1 µg/mL) for 12 h. Following the treatment, cells were divided into 3 groups for Western blotting analysis of cell cycle regulatory proteins, for flow cytometric analysis of cell cycle change, and for Hoechst staining of the nuclear morphological change. As shown in **Figure 6A**, co-treatment with CHX strongly suppressed nocodazole-induced early increase in cyclin B1 and Cdc2 levels. The suppression of nocodazole-induced early increase in cyclin B1 and Cdc2 protein levels by CHX was accompanied by a strong reduction in the population of G_2_/M-arrested cells (from 79.6 to 20.2%, flow cytometry analysis) (**Figure 6B** **, upper panel**) and abrogation of the development of prometaphase arrest (**Figure 6B** **, lower panel;** **Figure 6C**). Notably, treatment of cells with CHX alone did not appreciably alter the G_1_ phase cell population (**Figure 6B**). These observations further support the conclusion that increased *de novo* synthesis of cyclin B1 and Cdc2 contributes importantly to the development of prometaphase arrest in cells treated with nocodazole.

## Discussion

In a normal cell cycle, the transition from G_2_ phase to mitotic phase is triggered by the activation of the cyclin B1/Cdc2 complex. Cells with a suppressed cyclin B1/Cdc2 activity would be arrested in the G_2_ phase, whereas cells with an elevated cyclin B1/Cdc2 activity would tend to be favored to enter and proceed through mitosis [15]. The results of our present study confirm the earlier interesting observation showing that treatment of cancer cells with microtubule inhibitors such as vinblastine, colchicine and paclitaxel, causes a strong up-regulation of cyclin B1 and Cdc2 protein levels [9]–[12], while the level of Cdc2 phosphorylation at Tyr15 (an inactive form of Cdc2) was not significantly changed [23]. The observed up-regulation of cyclin B1 and Cdc2 is expected to result in increased functionality of the cyclin B1/Cdc2 complex. However, under these conditions, a higher percentage of cells are actually found to be selectively arrested in mitotic prometaphase; by contrast, control cells that are not treated with nocodazole and have much lower cyclin B1/Cdc2 levels actually have far fewer cells arrested in prometaphase. Apparently, these seemingly paradoxical changes are caused by the presence of nocodazole, which would create a false signal in prometaphase cells that they do not have adequate levels of cyclin B1/Cdc2 activity to proceed through mitosis. Consequently, cells arrested in prometaphase would increase their cyclin B1 and Cdc2 levels, as a cellular compensatory response to nocodazole treatment. As discussed below, the results of this study provide a series of experimental evidence in support of the notion that the strong, early up-regulation of cyclin B1 and Cdc2 following nocodazole treatment contributes critically to the development of prometaphase arrest as well as some of its unique features.

Earlier studies have shown that a rapid, excessive activation of the cyclin B1-dependent Cdc2 in G_2_ phase cells can result in aberrant entry into mitotic phase [29], [30]. Moreover, premature nuclear accumulation of the cyclin B1/Cdc2 complex will trigger chromosomal condensation and segregation [29], [30]. The results of our present study show that the marked early increase in cyclin B1 and Cdc2 levels is accompanied by rapid nuclear accumulation of these two proteins, in conjunction with the development of characteristic nuclear chromosomal condensation and segregation. In addition, we found that selective knockdown of cyclin B1 and Cdc2 strongly reduced the severity of nuclear chromosomal condensation and segregation as well as prometaphase arrest. Similar reductions in nuclear chromosomal condensation and segregation as well as prometaphase arrest were observed in nocodazole-treated cells when these cells were co-treated with roscovitine (an inhibitor of the cyclin-dependent kinases) or CHX (a protein synthesis inhibitor that reduces the levels of both cyclin B and Cdc2 in nocodazole-treated cells). Based on these experimental observations, it is reasonable to suggest that a stronger initial compensatory up-regulation of the cyclin B1/Cdc2 level following nocodazole treatment would result in severer prometaphase arrest because higher cyclin B/Cdc2 levels likely would bring about a severer degree of nuclear condensation and chromatin segregation. This suggestion is also supported by our observation that higher levels of cyclin B1 and Cdc2 were induced when higher concentrations of nocodazole were present, which causes a higher degree of microtubule inhibition and severer prometaphase arrest.

In this study, we found that knockdown of MAD2 almost completely abrogated nocodazole-induced up-regulation of cyclin B1 and Cdc2, suggesting that their up-regulation requires the presence of MAD2. At present, the mechanism by which nocodazole causes cyclin B1 and Cdc2 up-regulation is not understood. It is known that MAD2, acting through direct physical interaction with Cdc20 [31], [32], can inhibit the activity APC, a ubiquitin-protein ligase that tags proteins (including cyclin B1/Cdc2) for degradation [33]. We found that when the cells with MAD2 knockdown are treated with nocodazole, their Cdc20 protein level is essentially not altered while their cyclin B1 and Cdc2 up-regulation is completely abrogated (**Figure 4**). These observations suggest that a reduction in Cdc20-APC function likely is not the main reason for the observed strong up-regulation of cyclin B1 and Cdc2 in nocodazole-treated cells. In support of this suggestion, we also found that treatment of these cells with CHX almost completely suppresses the up-regulation of cyclin B1 and Cdc2 in nocodazole-treated cells, which clearly suggests that increased *de novo* protein synthesis is involved their up-regulation.

Roscovitine is a well-known inhibitor of the cyclin-dependent kinases. Interestingly, we found that treatment of cells with roscovitine also strongly suppresses nocodazole-induced up-regulation of cyclin B1 protein, although the mechanism of this suppression is not clear. Flow cytometric analysis of the cellular DNA content showed that the combined G_2_/M cell population is increased following treatment with roscovitine alone, but the population of prometaphase cells is only slightly increased. Our observations are in agreement with the earlier observation that roscovitine blocks MCF-7 cells in the G_2_ phase [21]. However, when roscovitine was used in combination with nocodazole, it strongly suppressed nocodazole-induced accumulation of the G_2_/M cell population as well as prometaphase cells. The dose-dependent effect of roscovitine in suppressing nocodazole-induced accumulation of both G_2_/M and prometaphase cell populations is closely correlated with its ability to suppress cyclin B1 up-regulation in nocodazole-treated cells. These data show that roscovitine, by inhibiting Cdc2 activity plus suppressing nocodazole-induced cyclin B1 up-regulation, inhibits cells from entering prometaphase by keeping them in the G_2_ phase when it is used together with nocodazole.

Co-treatment of cancer cells with CHX and nocodazole was also found to completely suppress the development of mitotic arrest. Since treatment of cells with CHX does appreciably affect the population of G_1_ phase cells (**Figure 6B**), the modulating effect of CHX on nocodazole-induced prometaphase arrest can be fully explained on the basis of its strong suppression of nocodazole-induced up-regulation of cyclin B1 and Cdc2 proteins. It is of note that the combination treatment of cells with CHX and nocodazole does not cause a stronger cell death; instead, it appears that these two agents can antagonize the cytotoxic effect of each other. This observation offers a mechanistic basis that these two classes of agents should not be used in combination in anticancer chemotherapy.

Interestingly, although flow cytometric analysis (based on measurement of cellular DNA content) shows that cells treated with nocodazole or roscovitine exhibit a similar G_2_/M cell cycle arrest pattern, the true nature of their cell cycle arrest is actually very different, based on the comparisons made in this study. While roscovitine predominantly induces G_2_ phase arrest (with minimal accumulation of prometaphase cells), nocodazole predominantly induces prometaphase arrest, along with a reduction in the G_2_ phase cell population. Similar to what we have observed with roscovitine, a selective knockdown of cyclin B1 and Cdc2 also results in a significant decrease in prometaphase cells, while it does not markedly affect the population of G_2_ phase cells. Based on the mechanistic explanations developed in this study, these results are reasonable because chemical inhibition or down-regulation of cyclin B1/Cdc2 will only block cells that have already completed DNA replication in the G_2_ phase from entering mitosis but they will not produce significant prometaphase arrest. Our observations are in agreement with this mechanistic explanation.

Nocodazole has been commonly used as a tool agent in cell biology to induce synchronization of the cell cycle at the G_2_/M phase. Based on the results of our present study, since this agent would predominantly produce prometaphase arrest (accompanied by aberrant chromosomal condensation and segregation resulting from extensive nuclear accumulation of cyclin B1 and Cdc2), this method of cell cycle synchronization may not be ideal for the purpose of studying normal cell cycles. In comparison, the use of a selective inhibitor of cyclin B1 or Cdc2 or the combined use of both inhibitors would seem to be a better approach given that these inhibitors will only accumulate G_2_ phase cells by slowing down the entry of G_2_ phase cells into M phase but will not cause severe prometaphase arrest and subsequent mitotic catastrophe as seen in cells treated with microtubule inhibitors.

**Figure 7:**
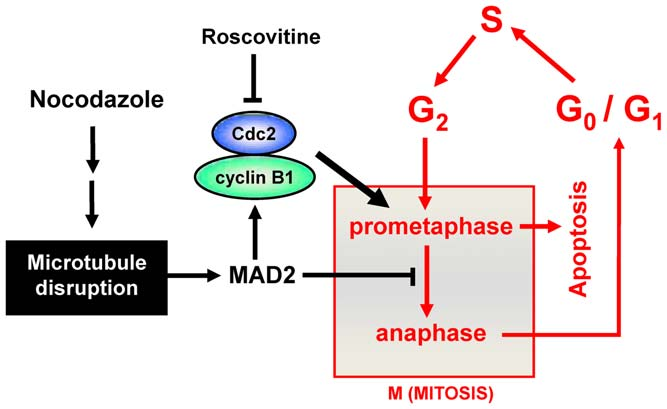
Schematic explanation of the contribution of an early cyclin B1/Cdc2 up-regulation to the development of prometaphase arrest in cells treated with nocodazole. Treatment of cancer cells with nocodazole causes microtubule disruption and thereby prevents microtubules from attaching to kinetochores in prometaphase cells. The unattached kinetochores will be bound by MAD2, which prevents the progression from prometaphase to metaphase and anaphase. It is speculated that the kinetochore-bound MAD2 protein plays an important role in mediating the up-regulation of cyclin B1 and Cdc2 proteins in prometaphase-arrested cells. The rapid increase of these two cell cycle proteins in prometaphase cells and particularly their accumulation in the nuclei are expected to be largely responsible for the development of characteristic nuclear phenotypes. Following a prolonged prometaphase arrest, the nocodazole-treated cells are expected to undergo cell death via intrinsic apoptosis pathways.

In summary, largely based on the findings made in the present study, the mechanistic explanation for the critical role of the cyclin B1 and Cdc2 up-regulation in nocodazole-treated cells in the development of characteristic prometaphase arrest is schematically depicted in **Figure 7**. The presence of nocodazole will cause disruption of microtubule formation in prometaphase cells, which subsequently results in failure of the microtubules to attach to kinetochores on the chromosomes. The unattached kinetochores are then bound by the spindle checkpoint protein MAD2. The binding of MAD2 at the kinetochores prevents the progression from prometaphase to metaphase and further to anaphase. It is speculated that the prometaphase arrest then creates a feedback up-regulation of cyclin B1 and Cdc2 protein levels, likely mediated through the kinetochore-bound MAD2 protein. The rapid rise of these two cell cycle proteins in prometaphase cells and particularly their accumulation in the nuclei are expected to be largely responsible for the development of characteristic nuclear phenotypes. Following a prolonged prometaphase arrest, the nocodazole-treated cells are expected to undergo cell death via intrinsic apoptosis pathways.

## Materials and Methods

### Reagents

Nocodazole, roscovitine, cycloheximide, and Eagle's minimum essential medium (EMEM) were obtained from Sigma, and fetal bovine serum (FBS) from HyClone (South Logan, UT). The antibiotics solution (containing 10,000 U/mL penicillin and 10 mg/mL streptomycin) was obtained from Invitrogen, and the trypsin-versene mixture (containing 0.25% trypsin and 0.02% EDTA) from Lonza Walkersville (Walkersville, MD). Anti-Cdc2 (Cdk1), anti-cyclin B1, and GAPDH antibodies were purchased from Cell Signaling Technology (Beverly, MA). Nocodazole was dissolved in 200-proof ethanol and stored at −20°C.

### Cell culture and MTT assay

MCF-7 and MDA-MB-435s human breast cancer cells and MCF-10A human mammary epithelial cells were obtained from the American Type Culture Collection (ATCC, Manassas, Virginia). The culture medium for MCF-7 cells was EMEM supplemented with 10% FBS, 2 µg/mL insulin, 0.5 mM sodium pyruvate, 10 mM nonessential amino acids, 2 mM L-glutamine, and antibiotics (100 units/mL penicillin and 100 µg/mL streptomycin). The medium for MDA-MB-435s cells was the Iscove's modified MEM containing 10% FBS and 3.024 g/L NaHCO_3_, and antibiotics. For culturing MCF-10A cells, a special mammary epithelium basal medium (MEBM, obtained from Lonza, Walkersville, MD) was used. This medium did not contain serum but was supplemented with an unspecified amount of epidermal growth factor, insulin, bovine pituitary extract, and hydrocortisone. These human cells were cultured at 37°C under 5% CO_2_, and were sub-cultured every 3 to 4 days. For determining cell viability, the 3-(4,5-dimethylthiazol-2-yl)-2,5-diphenyltetrazolium bromide (MTT) assay was used. MTT (10 µL, at 5 mg/mL) was added to each well at a final concentration of 500 µg/mL, and the mixture was further incubated for 1 h at 37°C, and the liquid in the wells was removed thereafter. DMSO (100 µL) was then added to each well, and the absorbance was read with a UV Max microplate reader (Molecular Devices, Palo Alto, CA, USA) at 560 nm. Unless indicated otherwise, the relative cell density usually was expressed as a percentage of the control group that was not treated with nocodazole.

### Crystal violet staining for estimation of cell number

To estimate the cell number, crystal violet staining was used as described earlier [34]. MCF-7 and MCF-10A cells were seeded in 96-well plates at a density of 5000 cells/well in cell culture media. At the end of the culture, the medium was discarded, and cells were fixed with 100 µL 1% glutaraldehyde at room temperature for 20 min. Glutaraldehyde was then discarded, and the cells were stained using 50 µL 0.5% crystal violet at room temperature for 15 min. After the crystal violet solution was discarded and rinsed three times with water, cells were solubilized in 100 µL 0.5% Triton X-100, followed by addition of 100 µL 200-proof ethanol. The UV absorbance at 560 nM was read on a UV max microplate reader (Molecular Device, Palo Alto, CA), and the value was used to reflect the relative cell number.

### Western blotting

For Western blotting, cells were washed first with phosphate-buffered saline (PBS) and then suspended in 100 mL lysis buffer (containing 20 mM Tris-HCl, 150 mM NaCl, 1 mM EDTA, 1% Triton X-100, 10 mL/mL protease inhibitor cocktail, pH 7.5). The amount of proteins was determined using the BioRad protein assay (BioRad, Hercules, CA). An equal amount of proteins was loaded in each lane, separated by 10% SDS-polyacrylamide gel electrophoresis (SDS-PAGE), and then electrically transferred to a polyvinylidene difluoride membrane (BioRad). After blocking the membrane using 5% skim milk, target proteins were immunodetected using specific antibodies. Thereafter, the horseradish peroxidase (HRP)-conjugated anti-rabbit IgG was applied as the secondary antibody, and the positive bands were detected using Amersham ECL Plus Western blotting detection reagents (GE Healthcare, Piscataway, NJ).

### Immunofluorescent microscopy

For immunocytochemical analysis, cells were first washed three times with PBS and fixed in 3% paraformaldehyde solution (3% paraformaldehyde, 0.1 mM CaCl_2_ and 0.1 mM MgCl_2_ in PBS, pH 7.4) for 10 min. Cells were then washed three times with PBS, permeabilized in 0.2% Triton® X-100/PBS for 5 min, and washed again three times with PBS. They were blocked with 10% normal goat serum (Jackson Immuno Research Labs, West Grove, PA) for 1 h, and washed with PBS. The cyclin B1 protein was detected using the cyclin B1 polyclonal antibodies (1∶100 dilution; Cell Signaling Technology), and Cdc2 protein was detected using the Cdc2 polyclonal antibodies (1∶100 dilution; Cell Signaling Technology). The first antibodies were incubated for 24 h at 4°C and followed by multiple washes in PBS. The same procedures were repeated with a fluorescein isothiocyanate (FITC)-conjugated secondary antibody (1∶200, Jackson Immuno Research Labs). The nuclei were stained with Hoechst-33342, and the coverslips were mounted on slides with Vectashield Mounting Medium (Vector Laboratories, Burlingame, CA). Fluorescein images were captured using a confocal fluorescence microscope (AXIO, Carl Zeiss Corporation, Germany).

### Flow cytometric analysis

After treatment with nocodazole, cells were harvested by trypsinization and washed once with PBS (pH 7.4). Cells were resuspended in 1 mL of 0.9% NaCl, and 2.5 mL of ice-cold 90% ethanol were added. After incubation at room temperature for 30 min, cells were centrifuged and the supernatant was removed. Cells were resuspended in 1 mL PBS containing 50 µg/mL propidium iodide (PI) and 100 µg/mL ribonuclease A and incubated at 37°C for 30 min. After centrifugation, cells were resuspended in PBS. Flow cytometric analysis was performed on a flow cytometer (model BD LSR II, BD Bioscience, San Jose, CA).

### Small interfering RNA (siRNA) treatment

The role of cyclin B1, Cdc2, and MAD2 in mediating nocodazole-induced cell cycle arrest was examined using siRNAs to selectively silence cyclin B1, Cdc2, and MAD2 genes. The cyclin B1 siRNAs (si-cyclin B1, catalog no. sc-29284, Santa Cruz), Cdc2 siRNAs (si-Cdc2, catalog no. sc-29252, Santa Cruz), MAD2 siRNAs (si-MAD2, catalog no. sc-35837, Santa Cruz), and the siRNA negative control (si-Con, catalog no. sc-37007, Santa Cruz) were obtained from Santa Cruz Biotechnology (Santa Cruz, CA). According to the supplier, each of the above siRNA preparations contains a combination of three target-specific RNA sequences that were designed to selectively knock down the expression of the corresponding target gene. MCF-7 cells were seeded the night before transfection, reaching a density of 30–50% confluence by the time of transfection. Forty nmol of si-cyclin B1, si-Cdcd2, si-MAD2, and si-Con were used for transfection using Lipofectamine 2000 (Invitrogen, San Diego, CA, USA) according to the instructions of the manufacturers. Transfected cells were maintained in culture for 2 days before harvesting and/or further analyses. The efficiency of the siRNA knockdown was determined by Western blot analysis.

### Statistical analysis

At least three separate experiments were performed for each measurement. All quantitative data were expressed as mean ± S.D. Comparisons between two groups were analyzed using two-way ANOVA. Individual differences among groups were analyzed using the Dunnett's test (SPSS-11.5 software). *P*<0.05 or *P*<0.01 was considered statistically significant or statistically very significant, respectively.

## Supporting Information

Figure S1 **Induction of cell cycle arrest and cyclin B1/Cdc2 up-regulation in MDA-MB-435s cells by nocodazole (Noco). A**. Time-dependent induction of mitotic arrest following treatment with nocodazole. MDA-MB-435s cells were seeded in 6-well plates at 5×10^4^ cells/mL and then treated with 250 nM nocodazole for 12 and 24 h. Cells were harvested and analyzed using flow cytometry (left panel). Cells were also stained with Hoechst-33258, and examined under a fluorescence microscopy (right panel) (at 100× magnification). As shown, many MDA-MB-435s cells are arrested in mitosis (prometaphase) after treatment with 250 nM nocodazole. **B**. Quantitation of the percentage of cells arrested in prometaphase (based on counting 200 or more nuclei in each sample) under a fluorescence microscope. Each bar is the mean ± S.D. from three measurements. * *P*<0.05 versus vehicle-treated controls. **C**. Time-dependent change in cyclin B1 and Cdc2 protein levels following nocodazole treatment. MDA-MB-435s cells were treated with nocodazole (250 nM) for 12 h, and total lysates were prepared. Western blots were detected using specific antibodies against cyclin B1 and Cdc2. Membrane was stripped for determining the levels of GAPDH as a loading control.
(TIF)

Figure S2 **Induction of cell cycle arrest and cyclin B1/Cdc2 activation in MCF-10A cells by nocodazole (Noco).** **A**. Both MCF-7 and MCF-10A cells were cultured in 96-well plates at 5,000 cells/well. Cells were incubated for 24 h allow for attachment. A time-dependent study was conducted with the intervals of 2, 3, and 4 days, respectively. The relative cell density was detected by crystal violet staining. **B**. MCF-10A cells were seeded in 6-well plates at 5×10^4^ cells/mL and then treated with 125 nM nocodazole for 12 and 24 h. Cells were stained with Hoechst-33258, and examined under fluorescence microscopy (at 200× magnification). **C**. Quantitation of the percentage of cells arrested in prometaphase (based on counting 200 or more nuclei in each sample) under a fluorescence microscope. Each bar is the mean ± S.D. value from three determinations. * *P*<0.05 versus vehicle-treated control. **D**. Cyclin B1 and Cdc2 protein levels following nocodazole treatment. MCF-10A cells were treated with nocodazole (125 nM) for the period as indicated, and total lysates were prepared. Western blots were detected using specific antibodies against cyclin B1 and Cdc2. Membrane was stripped for determining the levels of GAPDH as a loading control.
(TIF)
