# Supplementary figures and images for: Role of Cyclin B1/Cdc2 Up-Regulation in the Development of Mitotic Prometaphase Arrest in Human Breast Cancer Cells Treated with Nocodazole
Source: PLoS One. 2011 Aug 30;6(8):e24312. doi: 10.1371/journal.pone.0024312 (PMC3168870; doi:10.1371/journal.pone.0024312)

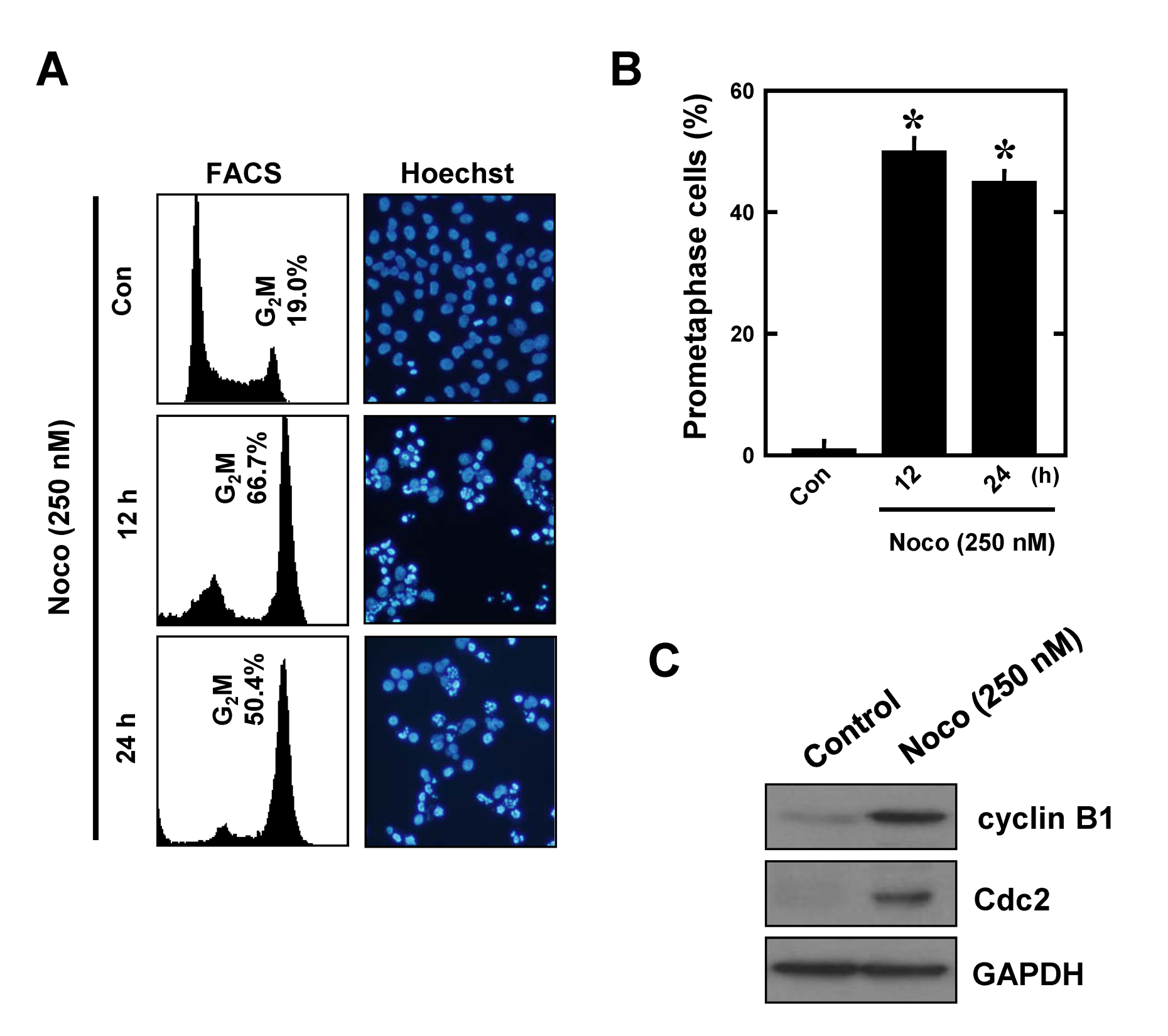

Supplement: Figure S1 — Induction of cell cycle arrest and cyclin B1/Cdc2 up-regulation in MDA-MB-435s cells by nocodazole (Noco). A. Time-dependent induction of mitotic arrest following treatment with nocodazole. MDA-MB-435s cells were seeded in 6-well plates at 5×10^4^ cells/mL and then treated with 250 nM nocodazole for 12 and 24 h. Cells were harvested and analyzed using flow cytometry (left panel). Cells were also stained with Hoechst-33258, and examined under a fluorescence microscopy (right panel) (at 100× magnification). As shown, many MDA-MB-435s cells are arrested in mitosis (prometaphase) after treatment with 250 nM nocodazole. B. Quantitation of the percentage of cells arrested in prometaphase (based on counting 200 or more nuclei in each sample) under a fluorescence microscope. Each bar is the mean ± S.D. from three measurements. * P<0.05 versus vehicle-treated controls. C. Time-dependent change in cyclin B1 and Cdc2 protein levels following nocodazole treatment. MDA-MB-435s cells were treated with nocodazole (250 nM) for 12 h, and total lysates were prepared. Western blots were detected using specific antibodies against cyclin B1 and Cdc2. Membrane was stripped for determining the levels of GAPDH as a loading control. (TIF) [file pone.0024312.s001.tif]

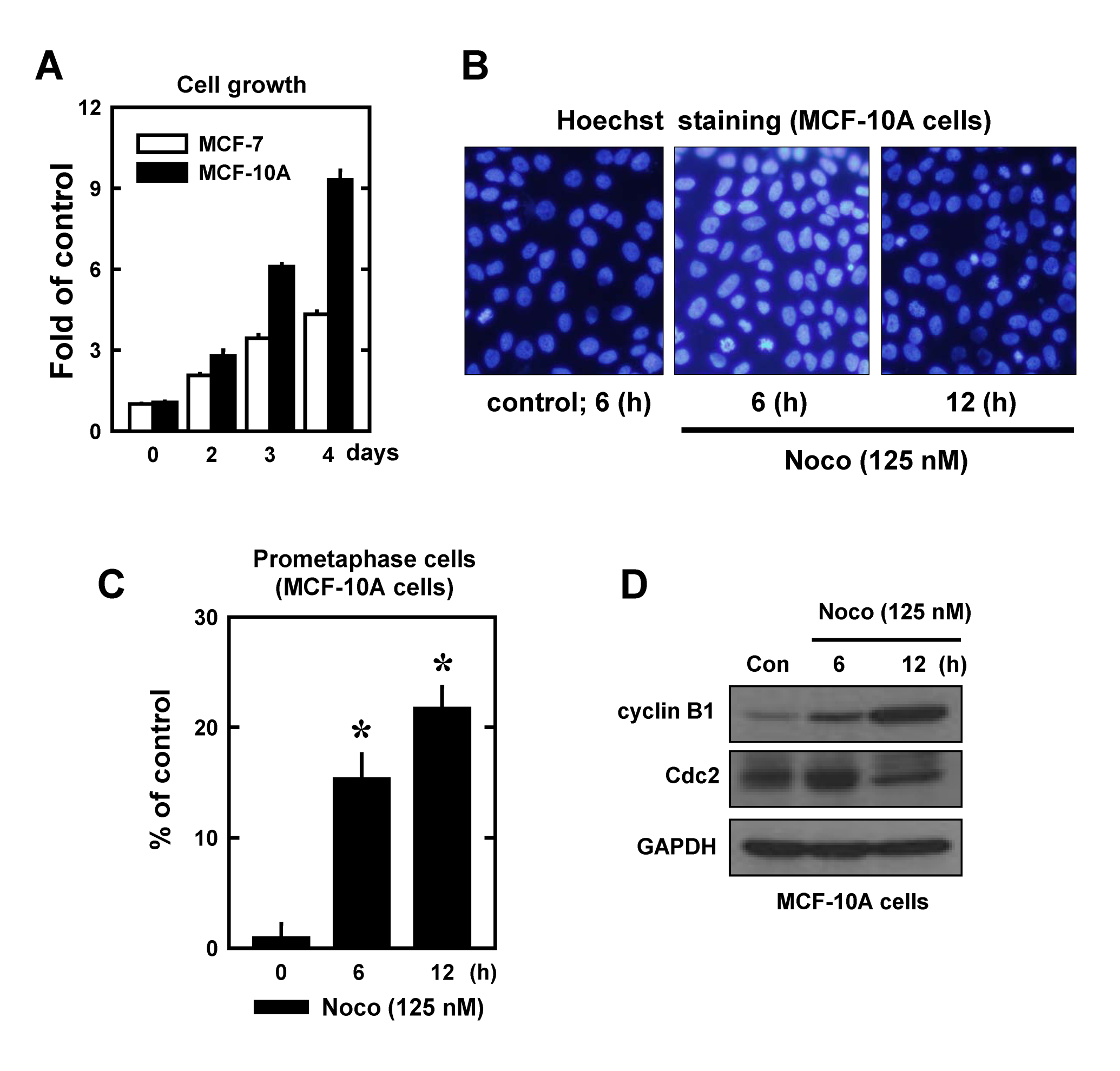

Supplement: Figure S2 — Induction of cell cycle arrest and cyclin B1/Cdc2 activation in MCF-10A cells by nocodazole (Noco). A. Both MCF-7 and MCF-10A cells were cultured in 96-well plates at 5,000 cells/well. Cells were incubated for 24 h allow for attachment. A time-dependent study was conducted with the intervals of 2, 3, and 4 days, respectively. The relative cell density was detected by crystal violet staining. B. MCF-10A cells were seeded in 6-well plates at 5×10^4^ cells/mL and then treated with 125 nM nocodazole for 12 and 24 h. Cells were stained with Hoechst-33258, and examined under fluorescence microscopy (at 200× magnification). C. Quantitation of the percentage of cells arrested in prometaphase (based on counting 200 or more nuclei in each sample) under a fluorescence microscope. Each bar is the mean ± S.D. value from three determinations. * P<0.05 versus vehicle-treated control. D. Cyclin B1 and Cdc2 protein levels following nocodazole treatment. MCF-10A cells were treated with nocodazole (125 nM) for the period as indicated, and total lysates were prepared. Western blots were detected using specific antibodies against cyclin B1 and Cdc2. Membrane was stripped for determining the levels of GAPDH as a loading control. (TIF) [file pone.0024312.s002.tif]
